# Dipeptidyl Peptidase-4 Inhibitor Use Is Not Associated With Acute Pancreatitis in High-Risk Type 2 Diabetic Patients: A Nationwide Cohort Study

**DOI:** 10.1097/MD.0000000000002603

**Published:** 2016-02-18

**Authors:** Chia-Hsuin Chang, Jou-Wei Lin, Shu-Ting Chen, Mei-Shu Lai, Lee-Ming Chuang, Yi-Cheng Chang

**Affiliations:** From the Institute of Preventive Medicine, College of Public Health, National Taiwan University (C-HC, S-TC, M-SL, L-MC), Department of Medicine, College of Medicine (C-HC, J-WL, L-MC, Y-CC), and Graduate Institute of Medical Genomics and Proteomics (Y-CC), Taipei, Taiwan; Department of Internal Medicine (C-HC, L-MC, Y-CC) and Center for Obesity, Life Style and Metabolic Surgery (Y-CC), National Taiwan University Hospital, Taipei, Taiwan; Cardiovascular Center, National Taiwan University Hospital Yun-Lin Branch, Dou-Liou City, Yun-Lin County, Taiwan (J-WL); Institute of Biomedical Science, Academia Sinica, Taipei, Taiwan (Y-CC).

## Abstract

To analyze the association between use of DPP-4 inhibitors and acute pancreatitis in high-risk type 2 diabetic patients.

A retrospective nationwide cohort study was conducted using the Taiwan National Health Insurance claim database. The risk associated with sitagliptin was compared to that with acarbose, a second-line antidiabetic drug prescribed for patients with similar diabetes severity and with a known neutral effect on pancreatitis. Between January 1, 2009 and December 31, 2010, a total of 8526 sitagliptin initiators and 8055 acarbose initiators who had hypertriglyceridemia or prior hospitalization history for acute pancreatitis were analyzed for the risk of hospitalization due to acute pancreatitis stratified for baseline propensity score.

In the crude analysis, sitagliptin was associated with a decreased risk of acute pancreatitis (hazard ratio [HR] 0.74; 95% confidence interval [CI]: 0.62–0.88) compared to acarbose in diabetic patients with prior history of hospitalization for pancreatitis or hypertriglyceridemia. The association was abolished after stratification for propensity score quintiles (adjusted HR 0.95; 95% CI: 0.79–1.16). Similar results were found separately in both patients’ histories of prior hospitalization of acute pancreatitis (adjusted HR 0.97; 95% CI: 0.76–1.24) and those with hypertriglyceridemia (adjusted HR 0.86; 95% CI: 0.65–1.13). No significant association was found for different durations or accumulative doses of sitagliptin. In the stratified analysis, no significant effect modification was found in relation to patients’ characteristics.

Use of sitagliptin was not associated with an increased risk of acute pancreatitis in high-risk diabetic patients with hypertriglyceridemia or with history of acute pancreatitis.

## Abstract 2

Supplemental Digital Content is available in the text

## INTRODUCTION

Dipeptidyl peptidase-4 (DPP-4) inhibitors are incretin-based therapies for type 2 diabetes mellitus. There has been a rapid global rise in their clinical use. DDP-4 inhibitors lower blood glucose by inhibiting the degrading enzymes of circulating incretins including glucagon-like peptide-1 (GLP-1) and gastric inhibitory peptide (GIP). DDP-4 exerts intermediate efficacy with a neutral effect on body weight and a low risk of hypoglycemia. International societies have recommend DPP-4 inhibitors and GLP-1 agonists as second-line treatment after metformin.^1^

However, in postmarketing surveillance, the US Food and Drug Administration (FDA) found a warning association between exenatide and reported acute pancreatitis cases.^2^ Similar cases were also reported in patients receiving sitagliptin. Therefore, the FDA revised the prescribing information for sitagliptin in 2009.^3^ The reported cases continued to increase and the concerns regarding the risk of pancreatitis and pancreatic cancer remained an ongoing debate. Several observational studies and clinical trials have reported inconsistent results^4–15^ and the FDA and European Medicines Agency have also announced ongoing efforts to assess the risk of acute pancreatic associated with incretin-based therapy.

However, 2 recently published systematic review and meta-analysis of randomized and nonrandomized studies suggested that incretins did not increase the risk of pancreatitis in general diabetic patients.^16,17^ In rodent models, exenatide and sitagliptin have been shown to increase inflammation of pancreatic acinar cells and the formation of intraepithelial neoplasia,^18–20^ while others reported that exenatide improved outcome of chemically induced pancreatitis.^21^ Data from the above studies suggest incretin-based therapy might exert differential effects on pancreatic inflammation in models with different backgrounds and exposure.

Since most clinical trials and observational studies either excluded or did not include sufficient number of type 2 patients at high risk of acute pancreatitis, the safety of DPP-4 inhibitors in this subgroup of patients deserves further studies. In this study, we specifically focused on high-risk diabetic patients for acute pancreatitis including those having prior hospitalization history for acute pancreatitis or those with hypertriglyceridemia. Since observational studies are often confounded by baseline difference in patients’ characteristics, we choose acarbose, a second-line antidiabetic drug prescribed in patients with clinical setting similar to those using sitagliptin and with neutral effect on acute pancreatitis as the reference. The hazard ratio (HR) of acute pancreatitis associated with sitagliptin compared with that of acarbose was estimated and was further stratified for propensity score.

## METHODS

### Data Source

The single-payer and compulsory National Health Insurance (NHI) program was implemented in Taiwan in 1995 and the enrollment rate reached over 99% by 2010. The Taiwan NHI Database includes complete outpatient visits, hospital admissions, prescriptions, disease, and vital status for 99% of the country's population (approximately 23 million). The current analyses linked several large computerized claims datasets with the National Death Registry through the use of birth dates and civil identification numbers unique to each beneficiary. The protocol was approved by the National Taiwan University Hospital Research Ethics Committee.

### Study Population

From the source population, we identified adult type 2 diabetic patients’ ages over 20 years who initiated sitagliptin or acarbose between January 1, 2009 and December 31, 2010. Initiation was defined as being free of study drugs for 12 months prior to the first prescription (index date). Acarbose was used as the active comparison group because it is used in settings similar to those of sitagliptin according to the guidelines and has not been associated with acute pancreatitis. Subjects were excluded if: they were 100 years of age or older, gender information was undetermined, they did not have continuous insurance coverage for 12 months before the index date, and they received both sitagliptin and acarbose on the index date. We restricted our study population to high-risk patients, defined as having prior hospitalization history for acute pancreatitis or receiving fibrates therapy for hypertriglyceridemia. Therefore, participants without prior history of acute pancreatitis or those who had never received fibrates therapy were excluded.

### Use of Study Drugs

The Taiwan NHI outpatient pharmacy prescription database recorded drug types, dosages, supply days, and total number of pills dispensed. The mean daily dose was calculated as total number of pills dispensed divided by the follow-up duration. The defined daily doses (DDD) were established by an expert panel as the typical maintenance dose required for its main indication in adults.

### Outcome Ascertainment and Follow-Up

The outcome of interest was the first hospitalization for acute pancreatitis, defined as having a discharge diagnosis of ICD-9-CM code 577.0. A previous validation study using a hospital administrative database reported a positive predictive value of 90% using this definition.^22^ Patients were followed from the index date to the earliest outcome occurrence, death or disenrollment from the NHI, or December 31, 2011.

### Covariate Ascertainment and Propensity Score Adjustment

Inpatient and outpatient diagnosis files and prescription files during the 12-month period before the index date were used to ascertain patients’ histories of ischemic heart disease, myocardial infarction, cerebrovascular disease, stroke, peripheral vascular disease, retinopathy, nephropathy, neuropathy, heart failure, atrial fibrillation, chronic kidney, liver, lung disease, depression, hypertension, dyslipidemia, any cancer, colorectal cancer, several gastrointestinal and pancreaticobiliary diseases including peptic ulcer disease, inflammatory bowel disease, gallstone disease, hospitalization history of acute appendicitis, diverticulitis, infectious diarrhea, and acute pancreatitis (ICD-9-CM codes provided in Supplementary Table 1). Information about concomitant medication use was also obtained, including insulin and other oral antidiabetic agents, antiplatelet agents, anticoagulants, antihypertensive agents, nitrates, statins, fibrates, digitalis, antiarrhythmic agents, cyclooxygenase-2 selective and nonselective nonsteroidal anti-inflammatory drugs (NSAIDs), proton pump inhibitors, histamine-2 receptor blockers, laxatives, systemic steroids, and antibiotics (anatomical therapeutic chemical [ATC] codes are provided in Supplementary Table 1). Demographic data of patient information included age, sex, and resource utilization (number of laboratory test measurements, abdominal ultrasounds, and colonoscopic examinations; number of outpatient visits; and number of hospitalizations) 12 months prior to the index date. Other information collected included monthly income as a proxy of socio-economic status.

All variables in Table 1 including demographic data, diabetes duration and related complications, other medical history, medications, and medical resource utilization were incorporated into a nonparsimonious logistic regression model. The probability of getting sitagliptin (i.e., the propensity score) was estimated and being used to adjust for the baseline differences between 2 treatment groups in the subsequent analyses.

**TABLE 1:**
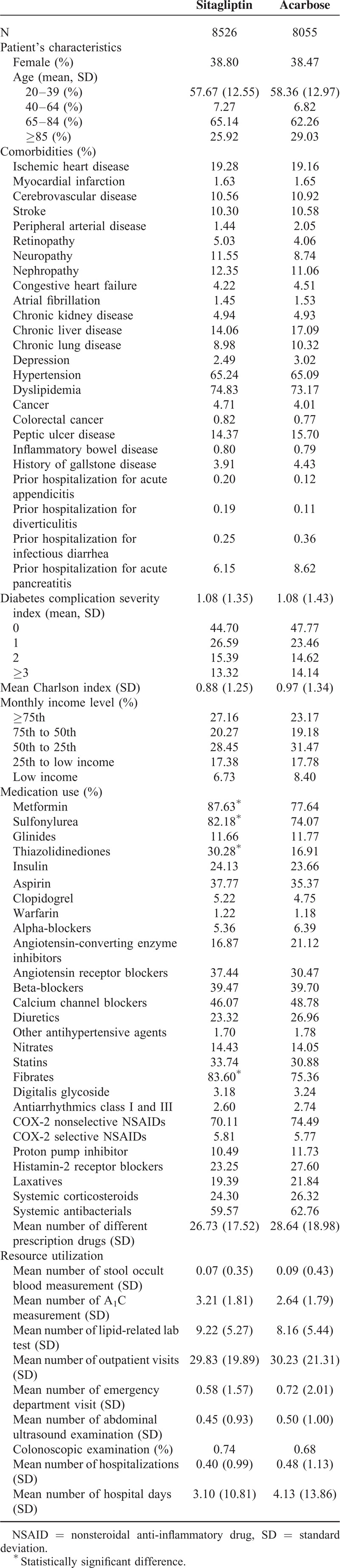
Baseline Characteristics Among Patients With Type 2 Diabetes and Hypertriglyceridemia or Prior History of Acute Pancreatitis Initiating Sitagliptin and Acarbose Therapy

### Statistical Analysis

Baseline characteristics, co-morbidities, medication use, and resource utilization among initiators of sitagliptin and acarbose were summarized. Person-days of follow-up in the 2 treatment groups were computed for all participants. The crude incidence rates for acute pancreatitis were calculated and their 95% confidence intervals (CIs) were estimated based on a Poisson distribution.

A Cox proportional hazards regression model stratified by baseline propensity score quintiles was used to calculate the HRs of hospitalization for acute pancreatitis and their 95% CIs using acarbose as the reference group.

To evaluate whether the risks became substantially elevated in the higher cumulative use groups, we classified cumulative dosage (≤180 DDD, >180 to ≤365 DDD, >365 to ≤545 DDD, and >545 DDD) and duration (≤0.5 year, >0.5 to ≤1 year, >1 to ≤1.5 years, and >1.5 years) of sitagliptin use into several predefined categories and used an extended Cox model with time-varying covariates for cumulative dosage/duration to calculate risk estimate for each category.

Stratified analyses were performed to evaluate potential effect modification. Participants were further stratified according to sex (men and women) and age (<60 and ≥60 years). A formal test for interaction was performed by using the likelihood ratio test. Two-sided *P* value <0.05 was considered to be statistically significant. All statistical analyses were performed with SAS 9.2 (SAS Institute, Cary, NC).

## RESULTS

**FIGURE 1:**
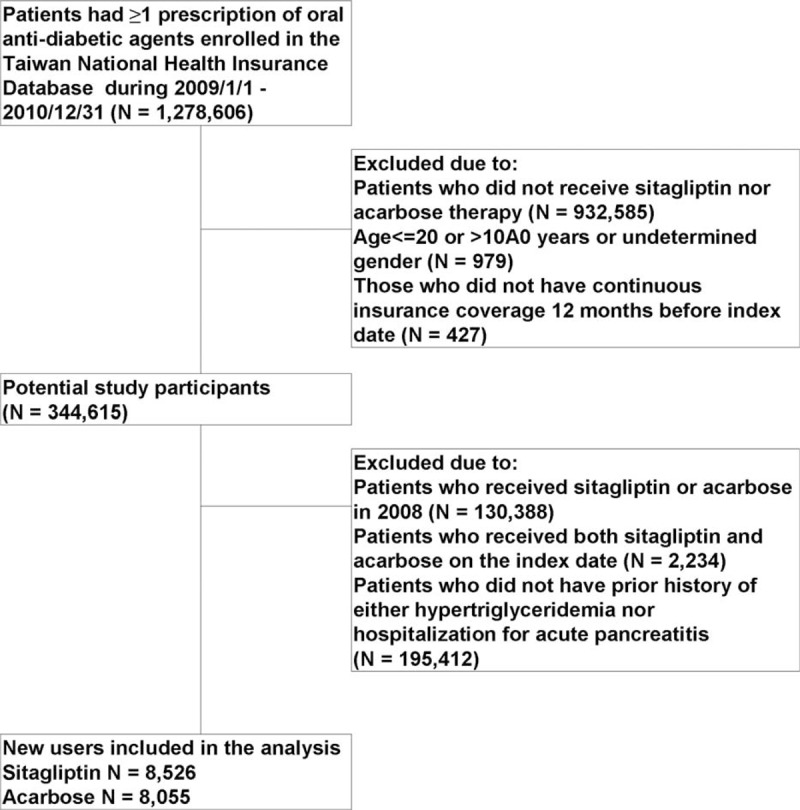
Flowchart of the study cohort assembly.

After excluding subjects who did not the meet study criteria, a total of 8526 sitagliptin initiators and 8055 acarbose initiators who had hypertriglyceridemia or prior histories of hospitalization for acute pancreatitis were included in the analysis (Figure 1). As shown in Table 1, these 2 treatment groups were similar in most baseline characteristics, including prior histories of several gastrointestinal and pancreaticobiliary diseases. However, a higher proportion of sitagliptin initiators also received other oral antidiabetic agents and fibrates therapy.

A total of 215 cases in the sitagliptin group and 282 cases in the acarbose group were hospitalized for acute pancreatitis acute during an average follow-up period of 1.9 years. As shown in Table 2, the crude incidence rate of acute pancreatitis per 100,000 person-day was 3.70 for sitagliptin initiators and 4.88 for acarbose initiators.

**TABLE 2:**
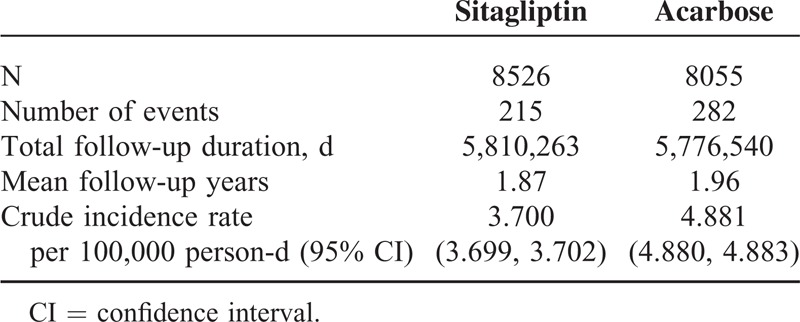
Person-Days and Crude Incident Rates of Acute Pancreatitis in Initiators of Sitagliptin and Acarbose

Table 3 presents results from the Cox regression model with acarbose as the reference group. In the crude analysis, sitagliptin was associated with a decreased risk of acute pancreatitis (HR 0.74; 95% CI: 0.62–0.88). In the analysis stratified by baseline propensity score quintiles, sitagliptin was not associated with a significantly higher risk (adjusted HR 0.95; 95% CI: 0.79–1.16). Similar results were found in patients with hypertriglyceridemia (adjusted HR 0.86; 95% CI: 0.65–1.13) and those with prior histories of hospitalization for acute pancreatitis (adjusted HR 0.97; 95% CI: 0.76–1.24).

**TABLE 3:**
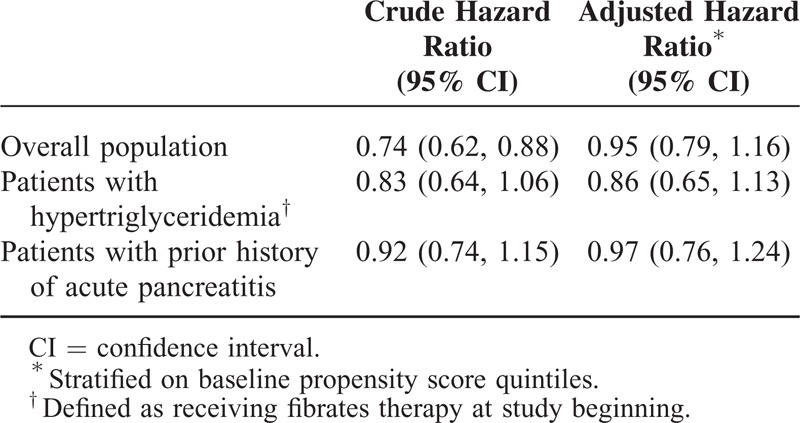
Hazard Ratios of Acute Pancreatitis Comparing Sitagliptin With Acarbose

In the analysis that evaluating the effect of duration of sitagliptin use on the risk of acute pancreatitis, we observe no increased risks associated with high cumulative use categories. The adjusted HR was 0.69 (95% CI: 0.42–1.15) for 1 to 1.5 years of use and 0.72 (95% CI: 0.34–1.52) for use >1.5 years (Table 4). There was also no higher risk for patients with cumulative dosage of 365 to 545 DDD (adjusted HR 0.85; 95% CI: 0.51–1.41) and >545 DDD (adjusted HR 0.69; 95% CI: 0.29–1.60).

**TABLE 4:**
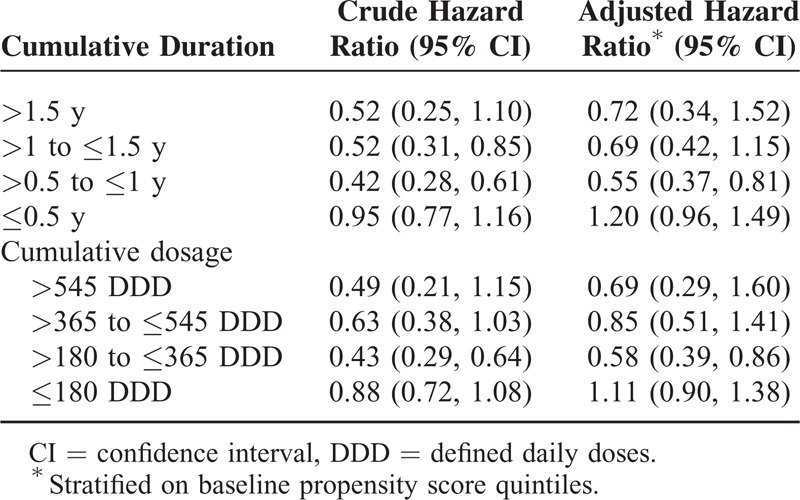
Hazard Ratios of Acute Pancreatitis Comparing Different Duration/Dosage of Sitagliptin Use With Acarbose

In the stratified analysis, no significant effect modifications were found in relation to patients’ characteristics. Similar risks were found for men and women, and patients ages <60 and ≥60 years (Table 5).

**TABLE 5:**
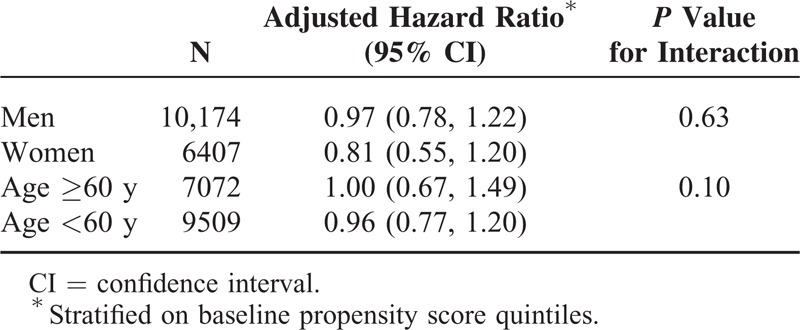
Adjusted Hazard Ratios of Acute Pancreatitis Comparing Sitagliptin With Acarbose Among Different Subgroups

## DISCUSSION

In this large nationwide cohort study, we analyzed the risks of acute pancreatitis associated with sitagliptin compared with acarbose in high-risk diabetic patients. No increased risk of acute pancreatitis associated with sitagliptin was found in either patients with prior histories of hospitalization for acute pancreatitis or histories of hypertriglyceridemia, even among patients with high cumulative use.

Since the first announcement of acute pancreatitis cases associated with exenatide and sitagliptin (or sitagliptin/metformin) by the US FDA,^2,3^ several reports from randomized clinical trials (RCTs) and observational studies have been published.^4–15^ Two RCTs of saxagliptin and alogliptin did not reported increased risk of acute pancreatitis.^14,15^ A recent meta-analysis pooling 134 RCTs revealed that use of DPP-4 inhibitors is not associated with acute pancreatitis (odds ratio [OR]: 0.93; 95% CI: 0.51–1.69).^18^ However, the total number of observed cases of incident acute pancreatitis is small (36 events). Furthermore, RCT tend to recruit patients with less co-morbidity, leading to a low incidence of acute pancreatitis than those in usual clinical setting. Therefore, current results from RCT are insufficiently powered to clarify the effect of DPP-4 inhibitor on acute pancreatitis. Another recent meta-analysis pooling 55 RCTs (n = 33,350) and 5 observational studies (n = 320,289) revealed that the incidence of pancreatitis among patients using DPP-4 inhibitors is low and these drugs do not increase the risk of pancreatitis (OR: 1.06, 95% CI: 0.46–2.45).^19^ The authors concluded that current evidence is not definitive and called for more carefully designed observational studies.

Large observational case–control or cohort studies reported inconsistent results. A study based on a US administration database found a 6-fold increased risk of acute pancreatitis associated with exenatide and sitagliptin compared to 4 other antidiabetic medications.^4^ A case–control analysis of 1269 patients and 1269 controls based on a large US administrative database found that use of exenatide or sitagliptin was associated with approximately 2-fold increased risk of hospitalization for acute pancreatitis.^5^

In contrast, a large US commercial health insurance database involving 16,276 sitagliptin users did not showed association with acute pancreatitis after follow-up for 1 year.^7^ A recent population-based case–control study from an Italian administrative database comparing 1003 cases admitted to hospital for acute pancreatitis and 4012 matched-controls also found no increased risk associated with incretins.^13^ Three following cohort studies based on different US health insurance claim databases^9–11^ and a cohort study based on 680 UK general practices^12^ also showed no excessive risk of incretin-based therapy (exenatide or sitagliptin) associated with acute pancreatitis.^12^ In another large cohort analysis of a US pharmacy claim database including 786,656 patients, the incidences of acute pancreatitis for nondiabetic controls, diabetic controls, exenatide, and sitagliptin diabetic users were 1.9, 5.6, 5.7, and 5.7 per 1000 patient-year, respectively. The risk of acute pancreatitis was significantly higher in the combined diabetic groups than in the nondiabetic controls. However, risk of acute pancreatitis was similar in the exenatide or sitagliptin users compared to diabetic control group, suggesting neutral effects of incretin-based therapy on acute pancreatitis in general diabetic patients.^8^ It should be noted that in subgroup analysis, the risk of acute pancreatitis is significantly increased in diabetic patients (relative risk [RR]: 2.1), patients with previous pancreatic disease (RR: 24.7), and patients with hypertriglyceridemia (RR: 1.4) compared with nondiabetic controls in this analysis,^8^ indicating the importance of controlling confounders such as the severity of diabetes and other risk factors of acute pancreatitis.

As previous studies have shown almost null, albeit inconsistent associations in general diabetic populations, here we focused on diabetic patients with high risk of acute pancreatitis. In a nested case–control study analyzing Taiwan NHI data, Chou et al^23^ reported significantly increased risks of acute pancreatitis among DPP-4 inhibitors users with hypertriglyceridemia (adjusted OR 1.80; 95% CI: 1.26–2.56) and pancreatic disease (adjusted OR 17.29; 95% CI: 10.60–28.19) as compared with nonusers, whom included a mixture of patients receiving a variety of antidiabetic agents.^23^ Since confounding by indication, especially the severity of diabetes, is a major bias in observational studies, we choose acarbose, a second-line antidiabetic drugs prescribed in clinical settings similar with sitagliptin and without known effect on pancreatitis as a comparator. Other comparators such as metformin or sulfonylureas tend to be prescribed earlier, while insulin tends to be used later in diabetic patients than DPP-4 inhibitors in Taiwan, leading to potentially biased baseline clinical characteristics. As shown in Table 1, the baseline characteristics of sitagliptin and acarbose initiators, including several gastrointestinal and pancreaticobiliary diseases, were very similar. Unexpectedly, in crude analysis, the HR of hospitalization due to pancreatitis associated with sitagliptin is significantly lower than that associated with acarbose. The reduced risk was abolished after further adjustment for propensity score using all baseline characteristics. Dose-dependent and duration-dependent analyses revealed a nonsignificant trend of lower incidence of acute pancreatitis in users treated with larger accumulative doses or longer durations.

Our results are consistent with a rodent study showing that exenatide treatment attenuated chemical-induced pancreatitis changes and release of amylase/lipase in normal rats and ob/ob mice.^17^ Several lines of evidence clearly demonstrated that incretin-based therapy prevented endoplasmic reticulum stress, reduce mitochondrial reactive oxygen species, and suppress inflammatory response of pancreatic beta-cell both in vitro and in vivo.^24–29^ Recently, the US FDA also performed its own pancreatic toxicology studies with exenatide in rodents including nondiseased controls, chemically induced pancreatitis, the Zucker diabetic fatty rat, and C57BL/6 mice fed a high-fat diet. Data from these studies did not identify exenatide-related pancreatic injury.^30^

The major strength of this study is the enrollment of a huge nationwide cohort of diabetic patients. Currently, this is the largest observational study looking for the association of DPP-4 inhibitors on acute pancreatitis in non-Caucasian population. The number of incident cases of acute pancreatitis is sufficiently large (497 cases) to clarify the association. Furthermore, our study extends the safety margin of incretin-based drugs to high-risk diabetic patients. The definition of acute pancreatitis by ICD-9 code used in our study has also been well-validated.

However, our study also suffers from several inherent limitations of observational studies. First, despite comprehensive propensity score adjustment, residual confounders still could not be excluded. Specifically, we did not have information related to other risk factors such as alcohol intake, smoking, and obesity, although these risk factors seldom influenced physician's prescription preference for sitagliptin or acarbose. Furthermore, we used propensity score method to incorporate a comprehensive list of baseline covariates to control for differences in characteristics between the 2 treatment groups. Second, because the vast majority of DPP-4 inhibitors used in this study were sitagliptin, thus our findings cannot be generalized to other DPP-4 inhibitors. Finally, although the follow-up time in this study is sufficient for the development of acute pancreatitis, subclinical low-grade pancreatic inflammation or pancreatitis attack not requiring admission to hospital cannot be detected.

In conclusion, in this nationwide cohort study with large number of incident cases in Taiwan, sitagliptin use seems not to be associated with increased risk of acute pancreatitis even in high-risk diabetic patients. These findings push the safety margin of incretin-based therapy to high-risk patients.

## Supplementary Material

Supplemental Digital Content
