# Supplementary material for: Dipeptidyl Peptidase-4 Inhibitor Use Is Not Associated With Acute Pancreatitis in High-Risk Type 2 Diabetic Patients: A Nationwide Cohort Study
Source: Medicine (Baltimore). 2016 Feb 18;95(7):e2603. doi: 10.1097/MD.0000000000002603 (PMC4998601; doi:10.1097/MD.0000000000002603)
Supplement: Supplemental Digital Content [file medi-95-e2603-s001.doc]

**Supplementary Table 1** ICD-9-CM codes and ATC codes used in this study

| **Comorbidities** | **ICD-9-CM codes** | **Medications** | **ATC codes** |
| --- | --- | --- | --- |
| Diabetes | 250 | Angiotensin receptor blockers | C09CA01, C09CA03, C09CA04, C09CA06, C09CA07, C09CA08, |
| Ischemic heart disease | 410-414 | ACE inhibitors | C09AA |
| Myocardial infarction | 410, 412 | Alpha-blockers | C02CA |
| Congestive heart failure | 428 | Beta-blockers | C07A |
| Cerebrovascular disease | 430-438 | Calcium channel blockers | C08 |
| Ischemic stroke | 433, 434, 436 (exclude 800, 801, 802, 803, 804, 850, 851, 852, 853, 854 ,V57) | Diuretics | C03 |
| Peripheral arterial disease | 440.2, 440.4, 443.81, 443.9 | Other anti-hypertensive agents | C02A, C02B, C02CC, C02D, |
| Retinopathy | 250.5, 362, 364.0, 364.4, 365-366, 368-369, 377 | Nitrate | C01DA |
| Nephropathy | 250.4, 580-588, 590, 593, 595, 596, 599, 791.0 | Insulin | A10A |
| Neuropathy | 250.6, 337.1, 354, 355, 356.8, 357.2, 358.1, 713.5, 729.2 | Sulfonylureas | A10BB |
| Chronic renal disease | 403.01, 403.11, 403.91, 404.02, 404.03, 404.12, 404.13, 404.92, 404.93, 585, V45.1, V56.0, V56.8 | Metformin | A10BA02 |
| Chronic liver disease | 070.2x, 070.3x, V02.61, 070.41, 070.44, 070.51, 070.54, V02.62, 571.0, 571.1, 571.2, 571.3, 571.4, 571.5, 571.6 | Thiazolidinediones | A10BG02, A10BG03 |
| Chronic lung disease | 490-496, 500-508 | Glinides | A10BX02, A10BX03 |
| Depression | 296.2,296.3,298.0,300.4,309.0,309.1, 293.83,296.90,309.28,296.82, 311 | Alpha-glucosidase inhibitors | A10BF |
| Hypertension | 401-404 | DPP4 inhibitors | A10BH |
| Dyslipidemia | 272 | Sitagliptin | A10BH01 |
| Colorectal cancer | 153, 154 | Saxagliptin | A10BH03 |
| Peptic ulcer disease | 531, 532, 533 | Vildagliptin | A10BH02 |
| Inflammatory bowel disease | 555, 556 | Statins | C10AA |
| Gallstone disease | 574, 575, 576.1 | Fibrates | C10AB |
| Acute appendicitis | 540 | Warfarin | B01AA03 |
| Diverticulitis/diverticulosis | 562 | Aspirin | B01AC06, N02BA01 |
| Infectious diarrhea | 001-009 | Clopidogrel | B01AC04 |
| *Clostridium difficle* infection | 008.45 | Cilostazol | B01AC23 |
| Acute pancreatitis | 577.0, 577.1, 577.2 | COX-2 nonselective NSAIDs | M01A (exclude M01AH, M01AX05) |
|  |  | COX-2 selective NSAIDs | M01AH |
|  |  | Digitalis | C01AA |
|  |  | Anti-arrhythmics Class I and III | C01B |
|  |  | Proton pump inhibitor | A02BC |
|  |  | Histamin-2 receptor blockers | A02BA |
|  |  | Laxatives | A06A |
|  |  | Systemic corticosteroids | H02AB |
|  |  | Systemic antibacterials | J01 |
